# Editorial

**Published:** 1974-07

**Authors:** 


### Editorial

This year the Bristol Medico-Chirurgical Society celebrates its Centenary. For a hundred years its members, drawn from the medical profession in Bristol and the surrounding West Country, have been meeting regularly for mutual edification, exchange of ideas and experiences and to benefit from the learning and wisdom of countless distinguished visiting speakers. From the start it had the closest links with the Bristol Medical School and its teaching hospitals but at the same time always welcomed the humblest practitioner on an equal footing with the most erudite professor. The part it has played in disseminating knowledge, maintaining professional standards and promoting friendship and understanding among members practising every branch of medicine cannot be overestimated. It created and gave to the University its first medical library. It founded this Journal which has a record of continuous publication since 1883 and will, we hope, celebrate its own centenary in nine year's time. The Society's contribution to Bristol's medical life is unique and it is a heritage we should treasure.

It is fitting that the Journal should commemorate this occasion in the life of the Society which gave it birth by the publication of this special number devoted to articles of an historical nature together with an account of the Celebration Dinner and a record of the names of those who, by holding office, have contributed so much to the Society's life over the first hundred years. They would rightly say that the undoubted success of the Society has been due to the loyal support of all its members. Long may this support continue and long may the Bristol Medico-Chirurgical Society flourish.

